# Why Is Mom Stressed: Homeorhesis as the Potential Problem and Nicotinamide Riboside as the Potential Solution

**DOI:** 10.1177/1179069519869679

**Published:** 2019-08-13

**Authors:** Charles Brenner

**Affiliations:** Department of Biochemistry, Carver College of Medicine, University of Iowa, Iowa City, IA, USA

**Keywords:** Postpartum, prolactin, lactation, nicotinamide adenine dinucleotide, brain-derived neurotrophic factor

## Abstract

The remodeling of female mammalian physiology to support the development of a fertilized egg into an externally breathing individual and then to provide all the nutrition to this individual while remodeling back to nearly her pregestational state is without parallel in male mammalian physiological transitions. While it is common parlance to refer to postpartum depression as a not infrequent stress in women, the postpartum physiological changes after every birth constitute profound metabolic stresses that are understudied and have important nutritional, behavioral, and neurodevelopmental implications for the maternal and neonatal health of every mammalian species. We discovered that the postpartum liver of a lactating female mouse has a depressed nicotinamide adenine dinucleotide (NAD) metabolome linked to circulation of higher levels of NAD metabolites in support of a >20-fold increase in NAD coenzymes in the mammary. Furthermore, by supporting a new mother’s apparent higher demand for NAD precursors, we increased circulation of prolactin, superinduced mammary biosynthetic programs, increased her time of arched-back nursing, enhanced mammary production of brain-derived neurotrophic factor, promoted postgestational weight loss, advanced the neurobehavioral development of her offspring, and allowed them to mature as stronger and more resilient adults with advantages in hippocampal neurogenesis and body composition. These results show that a new mother’s capacity for biosynthesis and functionally important nurturing is apparently limited by NAD. Here, we discuss homeorhetic flow of resources from a new mother to her offspring in the context of NAD metabolism and suggest avenues for future investigation.

**Comment on:** Ear PH, Chadda A, Gumusoglu SB, Schmidt MS, Vogeler S, Malicoat J, Kadel J, Moore MM, Migaud ME, Stevens HE, Brenner C. Maternal nicotinamide riboside enhances postpartum weight loss, juvenile offspring development, and neurogenesis of adult offspring. *Cell Rep*. 2019 Jan 22;26(4):969-983.e4. doi:10.1016/j.celrep.2019.01.007. PubMed PMID: 30673618. https://www.ncbi.nlm.nih.gov/pubmed/30673618

Biologists frequently cite homeostasis as the most remarkable feature of living things. The ability of living organisms to maintain their form and function over long periods of time is so remarkable that to, casual observation, living creatures may appear to violate the second law of thermodynamics by maintaining their organization over time.

No living creature violates the second law because no living creature is an isolated system. For example, the chipmunk I am watching in the backyard is in as good shape today as yesterday because it ate from my garden, whose biomass was created by solar energy and the incorporation of matter from the soil. Whereas homeostasis refers to the energy-dependent set of processes that allow us to repair and maintain ourselves, the larger process that incorporates the concept of continuous flow of energy (ie, from the sun to the chipmunk) is termed homeorhesis.

Just as there is a homeorhetic flow of energy from the sun to the strawberry to the chipmunk to its DNA repair, there is homeorhesis from a pregnant mother to her developing embryo and from a postpartum mother to her newborn. When the chipmunk is pregnant, its energy intake and resting metabolic rate are increased to support the development of the next generation of chipmunks. During the postpartum period, homeorhesis continues as the new mother marshals protein, fat, and carbohydrate for lactogenic calorie transfer to her offspring.^1^

Dale E. Bauman and W. Bruce Currie, pioneers in applying concepts of homeorhesis to maternal physiology, used dairy cows and sheep to show that provision of growth hormone and prolactin to mothers results in increased milk production and weight gain to their offspring.^2^ While the knowledge gained from these classical insights has been used in the livestock industry, little attention has been paid to the stresses homeorhetic processes exert on a mother during postpartum. In fact, people ask her when she is getting back to work and pretty much ignore the remarkable retransformation of her body after pregnancy—some of which is mediated by the process of breastfeeding and homeorhetic transfer to baby.^3^

Four nicotinamide adenine dinucleotide (NAD) co-enzymes [NAD^+^, reduced nicotinamide adenine dinucleotide (NADH), NAD phosphate (NADP^+^), and reduced NAD phosphate (NADPH)] are the central mediators of metabolism, essentially catalyzing the conversion of everything we eat into everything we are and everything we do.^4^ Quantitative targeted metabolomic technology^5^ has allowed us to discover that the NAD metabolome is dysregulated in multiple conditions of metabolic stress including obesity and type 2 diabetes,^6^ heart failure,^7^ diabetic and chemotherapeutic neuropathy,^8,9^ and central brain injury.^10^

One of the things that should be appreciated about the metabolic stresses that disturb the NAD system is that if a tissue’s NAD metabolites are tied up in a repair activity, then the tissue may be limited in the amount of fuel oxidation and/or anabolic processes that can be catalyzed. For example, if DNA is damaged and poly(ADP-ribose) polymerase is activated, thereby degrading NAD^+^, there is less NAD^+^ available for fuel oxidation. Similarly, if the hepatic NAD^+^ pool is largely reduced to NADH by ethanol intoxication or the hepatic NADPH pool is challenged with a storm of reactive oxygen species, one would expect that tissue to be quite stressed. This is clearly the case in heart failure,^7^ brain,^10^ and peripheral nerve^9^ injury in which particular metabolic stresses dysregulate the NAD metabolome.

The liver can be considered the most selfless organ in the body in the sense that it always does what is in the interest of other tissues, for example, glucose disposal after a meal but gluconeogenesis or ketogenesis in fasting states. Similarly, a new mother’s metabolism is self-sacrificing in that she mobilizes protein, fat, and carbohydrate for transmission to her offspring. In response to growth hormone and prolactin, the new mother transmits macronutrients from her adipose and liver to her mammary to support milk production.^11^ Thus, a new mother’s liver is the most selfless organ in the most selfless of creatures.

We therefore considered whether a new mother’s liver, the most self-sacrificing tissue in the most self-sacrificing creature, might be self-sacrificing not only in transmission of macronutrients but also in transmission of NAD metabolites. We discovered that at peak lactation (14 days after parturition), mouse mothers on healthy normal chow depress their liver NAD metabolome by ~1/3 and circulate a higher NAD metabolome in their blood by ~1/3 with respect to age-matched virgin females. Remarkably, at the same time, postpartum females have a mammary NAD metabolome that is expanded by 20 to 30-fold.^12^ When we supplemented new mother mice and rats with nicotinamide riboside (NR)^13^ at a level sufficient to boost their hepatic NAD metabolome against the postpartum flow of NAD metabolites from liver to mammary, they increased expression and circulation of prolactin by approximately 6-fold; superinduced blood and mammary NAD metabolomes; increased mammary size; superinduced mammary biosynthetic programs for protein, fat, and carbohydrate; and increased the amount of milk produced as well as the time of arched-back nursing.^12^

Not surprisingly, by boosting lactation, these mothers increased postgestational weight loss and produced pups that were larger at weaning.^12^ Unexpectedly, we found that the offspring of NR-supplemented mouse mothers are more adventurous in an open-field test, that weanling mice have better glycemic control if their mothers were NR supplemented, and that the weanling rats of supplemented mothers are learning-advantaged on a rotarod. Consistent with the enhanced adventurousness of young mice and enhanced physical learning of young rats, we found that the offspring of NR-supplemented mothers have advanced pruning in their caudate-putamen.^12^

Although the nutritional intervention only lasted for the 21 days in which new mothers nursed their pups, we discovered that the benefits of having a mother on NR last into mouse and rat adulthood. Specifically, we found that offspring of NR-supplemented mothers are less anxious, stronger, perform better on a beam walk, are more resilient to giving up in a forced swim, have better spatial memory, are advantaged in adult hippocampal neurogenesis, and have leaner body composition as adults.^12^

Although we found that NR-supplemented mothers transmit more milk to their pups and transmit a higher level of NAD precursor vitamins in their milk, we note that sacrificing pups to overfeed them does not produce healthier offspring.^14^ Instead, we hypothesized that there must be increased levels of bioactive factors in the milk of NR-supplemented mothers. Thus far, we discovered that the gene that encodes the brain-derived neurotrophic factor (BDNF) precursor is specifically upregulated in NR-supplemented mammary epithelium, that there is a higher level of BDNF in the milk of NR-supplemented mothers, and that there is a higher level of BDNF in the hindbrain of their offspring.^12^

The emerging picture is one in which a new mother is doing all she can for her new offspring in postpartum: nursing them, running her mammary biosynthetic programs for protein, fat and carbohydrate production, and depositing bioactives such as BDNF into the milk. These maternal activities are accompanied by a very significant redistribution of not only macronutrients but also NAD micronutrients from the liver to the mammary. And much like a hangover or a sunburn—both of which attack NAD homeostasis—can feel like a drain, we suspect that the mother’s homeorhetic transfer of resources may feel like a drain to her, such that restoring her hepatic NAD with NR gets her whole system to work better.

We are currently testing whether NR is uniquely capable of boosting maternal functions and performance of offspring or whether other NAD precursors have equivalent activities. We also aim to use omic methods to identify the range of bioactive factors that are upregulated by NR supplementation. We are keen to identify the mechanisms by which NR induces prolactin expression and how enhanced prolactin and mammary NAD metabolites are responsible for boosting the lactation program as well as the expression of specific bioactive factors such as BDNF. Although the oral availability of molecules such as BDNF is known,^15,16^ this is not well studied in this context, and there is nothing known about how dietary modulation of their expression could affect the development of offspring.

It has not escaped our notice that postpartum lactating women, particularly in conditions of suboptimum nutrition or other types of metabolic stress, might benefit from specific nutritional interventions that address the homeorhetic transfers we have characterized. Thus, the identification of mechanisms and biomarkers from animal research has the potential to help us design clinical evaluation of safe molecules that will address postpartum metabolic stress, increase lactation, and potentially aid postgestational weight loss and neonatal development in human populations.
